# Carbohydrate Elimination or Adaptation Diet for Symptoms of Intestinal Discomfort in IBD: Rationales for “Gibsons' Conundrum”

**DOI:** 10.1155/2012/493717

**Published:** 2012-02-29

**Authors:** Q. Manyan Fung, Andrew Szilagyi

**Affiliations:** Division of Gastroenterology, Department of Medicine, Jewish General Hospital, McGill University School of Medicine, 3755 Côte-Sainte-Catherine Road, Room E110, Montreal, QC, Canada H3T 1E2

## Abstract

Therapeutic use of carbohydrates in inflammatory bowel diseases (IBDs) is discussed from two theoretical, apparent diametrically opposite perspectives: regular ingestion of prebiotics or withdrawal of virtually all carbohydrate components. Pathogenesis of IBD is discussed connecting microbial flora, host immunity, and genetic interactions. The best studied genetic example, NOD2 in Crohn's disease, is highlighted as a model which encompasses these interactions and has been shown to depend on butyrate for normal function. The role of these opposing concepts in management of irritable bowel syndrome (IBS) is contrasted with what is known in IBD. The conclusion reached is that, while both approaches may alleviate symptoms in both IBS and IBD, there is insufficient data yet to determine whether both approaches lead to equivalent bacterial effects in mollifying the immune system. This is particularly relevant in IBD. As such, caution is urged to use long-term carbohydrate withdrawal in IBD in remission to control IBS-like symptoms.

## 1. Introduction

A conundrum is defined by the American Heritage Dictionary of the English language [1] as “a riddle, especially one whose answer makes a play on words or as a puzzling question or problem.” In 1995, Gibson and Roberfroid published their treatise on the potential benefits of maldigested carbohydrates on host health through manipulation of microflora [2]. The concept of prebiotics (nondigestible, highly fermentable, dietary substances that exhibit beneficial functions in the host by facilitating the growth and metabolic activity of either one or a selective number of health-promoting colonic species) coincided with the emergence of potential human benefits found in probiotics (live bacteria bypassing the acid environment of the stomach and conferring health benefits to the host. A combination of pre- and probiotics is referred to as a synbiotic). A deluge of basic and clinical studies ensued as well, on the effects of prebiotics on an array of diseases. In particular, Crohn's disease (CD) and idiopathic ulcerative colitis (UC) (the two clinical subtypes of IBD) were targeted to capitalize on the potential therapeutic effects of either pro- or prebiotics [3–5]. While CD and idiopathic UC both share somewhat similar epidemiology and are thought to have originated from common genetic and environmental etiogenesis, they are in fact considered as two different entities. CD is unrestricted to any part of the gastrointestinal tract, in which the terminal ileum with or without the proximal colon remains the most common site affected. In UC, pathology tends to begin in the distal rectum and then it may proceed to involve the rest of the colon in a uniform fashion.

**Figure 1:**
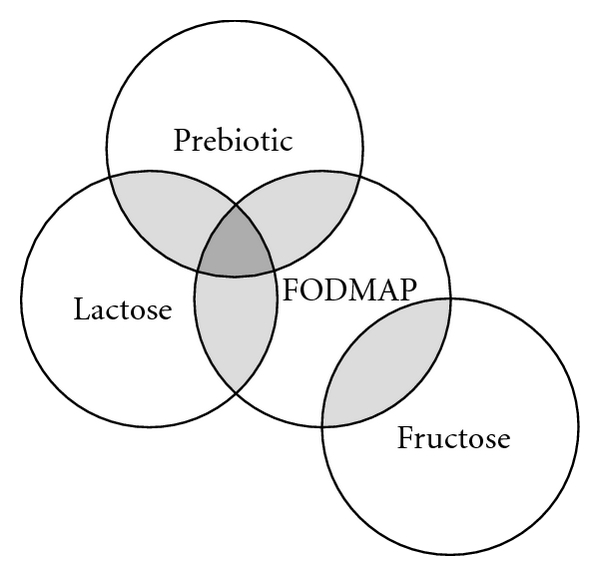
This Venn diagram shows the relationship between FODMAP, comprises of fructose, oligosaccharides, disaccharides, monosaccharides, and polyols. The central diet includes the majority of carbohydrates which are hypothesized to be malfermented by lower intestinal bacteria and therefore leading to excess production of gas and short-chain fatty acids with induction of symptoms. Thus, FODMAP includes all prebiotics in which lactose is included also as a restricted probiotic in lactose maldigesters. It is the hypothetical benefits of either withdrawal from diet or adapting to the prebiotic components of this diet that potentially forms a scientific conundrum in application.

Similarly a benign but lifestyle-altering condition of irritable bowel syndrome (IBS—a chronic functional bowel disorder encompassed by frequent recurrences of abdominal pain is associated with altered bowel movements: diarrhea, constipation, or alternating form) also fell into the category potentially ameliorated by probiotics and perhaps prebiotics. In both of these conditions, however, it was postulated that bacterial interactions, abnormal fermentation, and host handling of fermentative products as well as an immune response rather contributed to aggravation of symptoms [6, 7]. In 2005, Gibson and Shepherd hypothesized such mechanisms in causation of gastrointestinal symptoms in these disorders and suggested that carbohydrates be withdrawn from diets of symptomatic IBS or IBD patients. This FODMAP diet suggests the withdrawal of fermentable oligo-, di-, monosaccharides, and polyols from the diet [8]. As such, the FODMAP diet includes lactose and most other prebiotics (refer to Figure 1 and Table 1). Some of these recommendations, of careful carbohydrate selection for diet in patients with IBD, were also suggested earlier in a book by Gottschall [9]. There was less emphasis on small molecules except for sweeteners and more on large complex carbohydrates.

The presentation of these two hypotheses, then, formulates a conundrum. In the first instance, carbohydrates bypassing absorption in the small intestine can specifically manipulate metabolism and benefit the commensal bacteria, which in turn help reduce inflammation. It is important to note that there are specific recognized prebiotics, but all carbohydrates interact with the microbiome. In the second scenario, a wide array of carbohydrates, including prebiotics, are withdrawn. Are the end results equivalent, that is, has a stimulated immune system been placated? A similar paradigm applies to use of probiotics [10] or antibiotics which have received some success both in IBD [11] and IBS [12–14] as well as to other dietetic interventions. These include enteral/polymeric diets and nonspecific exclusion diets that have previously been implicated for their therapeutic roles which are beyond the scope of this review [15].

Herein we will focus on discussing the rationales behind the usage or nonusage of FODMAP diet in IBD. Effects in IBS will be discussed overall, but the concept will be discussed in detail as it might apply to IBD based on current concepts of pathogenesis. The objectives are to review carbohydrate pathogenic interactions with intestinal immunity and to conceive an effective intervention that convenes the apparent hypothetical contradictions inherent in the two approaches to carbohydrate use.

## 2. Microbial Diversity in the Gut

### 2.1. Normal Development

The gastrointestinal microbiota (or microflora) differs among individuals and its dominant bacterial phylotypes are acquired from the moment of birth. Although intestinal microbial composition will remain fairly constant from early infancy throughout adulthood once bacterial colonization is established [16], these microorganisms respond adaptively to better accommodate and protect at an individual level. As such, fecal samples adequately reflect the colonic bacterial environment and indicate the individual's intestinal status in response to developmental changes, environmental factors, antibiotic usage, or illness. The recent advancement in molecular profiling methods, such as high-throughput sequencing of microbial 16S ribosomal RNA genes [17] and metagenomics [18], provides a comprehensive insight into the 100 trillion bacteria that currently comprise the microbiota in the distal gastrointestinal tract alone. Representing the two predominant phylotypes found in mucosal and luminal microbiota are Gram-positive Firmicutes (species including Clostridia and Lactobacillaceae) and Gram-negative Bacteroidetes (species including Bacteroides), all of which are obligatory anaerobic bacteria.

### 2.2. Functions of the Microbiome

Sharing a symbiotic relationship with a dynamic bacterial community also means acquiring a diverse metabolic profile essential for intestinal development [19]. The resident microflora promotes the differentiation and proliferation of enteric epithelial cells by harvesting essential minerals (e.g., iron, calcium, and magnesium) as well as mediating the synthesis of vitamins (e.g., cobalamin, vitamin K, biotin, pyridoxal phosphate, and tetrahydrofolate). In addition, the bacterial genome (also known as the microbiome) encodes a large repertoire of saccharolytic enzymes, including glycoside hydrolases and polysaccharide lyases, needed to further metabolize nondigestible carbohydrates such as plant polysaccharides (dietary fibers), oligosaccharides, lactose (especially in lactose maldigesters), and sugar alcohols in the proximal colon through a process called saccharolytic fermentation [18]. This colonic fermentation of macronutrients yields various end products like gaseous compounds (e.g., hydrogen gas, methane, and carbon dioxide) and short-chain fatty acids (SCFAs), with the latter being mostly comprised of acetate, propionate, and butyrate. These are utilized as the primary energy source for the colonic mucosa. Colonic concentration of SCFA substrates is determined not only by the consumption of dietary fiber but also by the bacterial species present in the microbiota. For example, the two prominent bacterial phylotypes each differs in the types of SCFAs produced, with Firmicutes selectively producing butyrate and Bacteroidetes controlling the levels of acetate and propionate production [20, 21]. Small carbohydrates like lactose may also lead to production of butyrate through the stimulation of second tier bacteria (butyrogens) by initial breakdown products [22, 23]. The presence of these organic acids helps induce an acidic environment unfavourable for the proliferation of strict anaerobic species [24, 25]. Once carbohydrates are no longer available for fermentation, bacteria will proceed to proteolytic fermentation (less favourable) in the distal colon where proteins derived from diet, endogenous cellular proteins, and bacterial cells are catalyzed to toxic, carcinogenic metabolites (e.g., bacteriocins, ammonia, indoles, and phenols) [26]. These substances inhibit the growth or kill potentially pathogenic constituents. Another way the microbiota maintains resistance against colonization by pathogenic organisms is to compete for nutrients and attachment sites to the mucosal surface in the colon [27, 28]. Minor perturbations in the intricate microbial diversity can have significant impact on the gut homeostatic balance [29–32]. These changes have been implicated to predispose or contribute to conditions such as sepsis, IBS symptoms [6, 33], and even obesity in some populations [34, 35].

## 3. The Interaction of the Microbiome with Intestinal Mucosal Immunity

### 3.1. Mucosal Immune System

Intestinal mucosal immunity is associated with the integrity of the intracellular junctions in the gut epithelium constituting what is called a physical barrier. The mucosal integrity is further strengthened by what is called a chemical barrier thanks to a specialized group of differentiated epithelial cells residing in the paracellular space. Goblet cells, for instance, are responsible for secreting an overlying glycocalyx layer composed of mucin glycoproteins [36]; production of defensins, immunoglobulins, and other substances by enterocytes, lymphocytes, and Paneth cells (the last being generally restricted to the crypts of Lieberkuhn in the distal small bowel) can also be found within this mucus layer [37, 38]. This dual barrier provides enhanced protection against unwarranted entry of luminal contents (including self- and non-self-antigens) into the systemic immune system, but this is also where innate immune recognition takes place. Many of the cells in this mucosal barrier respond to pathogens by expressing two functionally important subsets of pattern-recognition receptors (PRRs)—extracellular Toll-like receptors (TLRs) and intracellular nuclear oligomerization domain-(NOD-) like receptors. These assist in the detection of pathogen-associated molecular patterns (PAMPs) through the leucine-rich repetitive (LRR) domain. Lipopolysaccharides (LPSs) and peptidoglycan (PGN) components (i.e., muramyl dipeptide) of bacterial cell wall are two examples of PAMPs. Each subset can either individually or convergently activate nuclear factor *κ*B (NF-*κ*B) effector in the defense against foreign pathogens by producing inflammatory cytokines (e.g., TNF-*α* and IL-1*β*) and antimicrobial peptides [39]. Chronic stimulation of PRRs by PGN can also produce inhibitory cytokines (e.g., TGF-*β* and IL-10) via the NOD2-dependent pathways to minimize excessive tissue injury induced by intestinal antigen-presenting cells [40]. Intestinal mucosal immunity is reinforced further by continuous interaction between epithelial cells and adaptive immune cells, including effector T-helper cells (Th1, Th2, and Th17), regulatory T cells (Foxp3+ Treg), and other immune cells (i.e., dendritic cell, macrophage, and natural killer cell) at the follicle-associated epithelium junction overlying the gut-associated lymphoid tissue [41].

Central to the discussion in conferring protection to the host is the influences of microbiota community on the normal development and homeostasis of mucosal immunity [52–54]. The symbiotic nature of the host-microbiota relationship is fundamental to the shaping of immunological function, balance, and tolerance in the gut. Paradoxically, the key for preserving such symbiotic coexistence in return depends on the robustness of the intestinal immune network, particularly in its ability to differentiate between symbiotic and pathogenic colonization. The maintenance of gut homeostatic balance, therefore, depends on the cooperation between mucosal immunity and microbial community, that is, if the right microbiota composition is present. Alteration to the microbial ecology, commonly referred to as dysbiosis, can distort intestinal immune responses by shifting the equilibrium between pro- and anti-inflammatory T-helper cells differentiation, as characterized by IBD pathogenesis (Box 1) [55–57].

### 3.2. Concept of Dysbiosis and IBD

Analyses in the gastrointestinal microbial populations showed significant differences between healthy individuals and patients with IBD, an indication that dysbiosis may be a contributing factor to IBD [58, 59]. Specifically, an increased propensity of obligatory aerobic bacteria is seen displacing the anaerobic species, with Bifidobacteria (in CD) [60] and Lactobacilli (in UC) [20] both being deficient in the microbiota. Reduced diversity of mucosa-associated phyla Firmicutes and Bacteroidetes is commonly observed as well in IBD controls [59, 61]. Depletion of *Faecalibacterium prausnitzii* is related to an activated immune response, which specifically suppresses and eradicates selective groups of bacteria resulting in an imbalance of intestinal flora [62]. This is relevant due to *F. prausnitzii* belonging to the genus Firmicutes in the Clostridia 14 cluster, which in fact is an important butyragenic-stimulated bacterium capable of exerting anti-inflammatory effects [63]. 

Swidsinski et al. has shown that a person afflicted with IBD displays an intestinal mucosa heavily populated with adherent organisms which are virtually nonexistent in a healthy individual [64]. For instance, adherent-invasive E. coli are isolated and found to adhere to the brush border of primary ileal enterocytes of CD patients but none in healthy controls [65–67]. Most recently, however, Willing et al. demonstrated that specific bacterial changes were associated with different anatomical sites in CD but UC patients in remission shared a similar microflora as to healthy controls [68]. This correlates with the data gathered from a comparative microbiota analysis of mice where they found closely related phylotypes displayed higher abundances (cooccurrence) and are conducive to intestinal colonization irrespective of the microbial origin (external or internal) [69]. Highly abundant subsets of commensal microorganisms, such as *Helicobacter*, *Clostridium*, and *Enterococcus *species, are hence more susceptible to transform the symbiotic nature of the host-microbiota relationship into a pathogenic one under certain environmental conditions [54]. Mucosal antibodies recovered from IBD subjects are found to be directed against intestinal commensal bacteria, as such, they may be more responsive to antibiotic treatment and faecal diversion than non-IBD controls [70, 71].

## 4. The Relationship of Bacterial Metabolites of Carbohydrates and Mucosal Immunity

### 4.1. Immunoregulatory Functions of Short-Chain Fatty Acids

Given that environmental-induced changes can alter the intestinal microbiota, leading to dysregulatory inflammatory responses, increasing evidence indicates that microbial fermentative by-products (e.g., acetate, propionate, and butyrate) demonstrate anti-inflammatory properties that may be clinically relevant to the treatment of IBD [14, 77, 90–93]. One study attributed the interaction between acetate and the chemoattractant receptor, G-protein-coupled receptor 43 (GPR43; also referred to as FFAR2) [94], critical in the regulation as well as the resolution of inflammatory responses [95]. By analyzing the transcription profiles of cellular receptor genes found in human leukocytes, the investigators had identified high degree of GPR43 expression in neutrophils and eosinophils; its expression was also closely governed by Toll-like receptors (TLR2 and TLR4), formyl peptide receptors (FPR1 and FPR2), and C5aR suggesting that GPR43 is important for innate immune and chemoattractant-induced responses. To examine the anti-inflammatory protection conferred by the acetate-GPR43 signalling pathway, they induced acute colitis by adding dextran sulphate sodium (DSS) to the drinking water of GPR43-deficient (*Gpr43^−/−^*) and wild-type mice for one week. Compared to the wild-type, *Gpr43^−/−^*mice exhibited exacerbated inflammatory response based on histological analysis, daily activity index (DAI; a combined measure of weight loss, rectal bleeding, and stool consistency), and increased levels of myeloperoxidase activity (MPO; inflammatory mediator) in the colon. A significant improvement to those inflammatory parameters soon followed after 200 mM acetate was introduced in their drinking water in a GPR43-dependent manner (*Gpr43^−/−^* mice lacked the receptor to respond to acetate but not in wild-type ones). Similar development of unresolved inflammation occurred in other mice models such as DSS-induced colitis in germ-free wild-type, K/BxN serum-induced model of inflammatory arthritis and ovalbumin-induced model of allergic airway inflammation. Host protection against enteropathogen *Escherichia coli* (0157:H7) infection was recently linked to acetate production by Bifidobacteria [96]. They proposed that acetate prevented the pathogen from entering the systemic circulation by enhancing mucosal barrier defense.

Although acetate and propionate have long been shown to exert immunologic modification [14], it is butyrate which generates the majority of interest in research. The immunoregulatory activities exerted by butyrate are listed in Table 2. Butyrate is able to regulate multiple gene expressions in the colonic epithelial cells [74, 75]. Inhibition of histone deacetylase by butyrate has been identified to orchestrate a series of downstream effectors responsible for its attributive anti-inflammatory profile [76, 97]. Most notably is the direct suppression of the NF-*κ*B transcription factor via histone acetylation, which in turn alters the transcriptional patterns of many genes encoding cytokines, chemokines, adhesion molecules, and other proinflammatory mediators [77, 98–101]. 

Other anti-inflammatory properties of butyrate highlighted as possible therapeutic targets in IBD include its ability to modulate an intracellular JAK/STAT1 signalling cascade which reduces NO production in macrophages and in intestinal myofibroblasts [79]; enhance the upregulation/detection of PRRs on intestinal epithelial cells (e.g., TLR1, TLR4, TLR6, peroxisome proliferator-activated receptor-*γ* (PPAR*γ*)) [80–83], hence facilitating the migration of neutrophil [102]; mitigate the extent of DNA damage in colonocytes induced by neutrophilic oxidizing species during carcinogenesis [103, 104]; potentiate the expression of heat shock proteins, especially HSP70 and HSP25, in enterocyte-like Caco-2 cells and DSS-induced colitis which further enhances cellular protection during an inflammatory response [105, 106].

In some cultured cell lines, butyrate improved the status of intestinal defense mechanisms commonly impaired in IBD by restoring mucosal barrier integrity and promoting epithelial migration in a dose-dependent manner [87–89]. Specifically, its administration has been demonstrated to stimulate MUC2 mucin gene expression in which its protein product is often altered in IBD [107–109]. An increased mucin secretion has also been reported in the isolated vascularly perfused rat colon [110]. Butyrate was also demonstrated to modulate the expression of antimicrobial peptide, cathelicidin (LL-37), in isolated colon epithelial cell lines [111]. A reinforced mucus layer and epithelial tight junctions mean decreasing mucosal permeability, making foreign substances impossible to pass through the defense barrier.

To date potential therapeutic effects of butyrate have been limited to UC. Interestingly, in vivo studies have shown that butyrate oxidation in the colon mucosa of patients with quiescent UC remain normal, whereas those with an actively inflamed mucosa do not [112]. It was reported that TNF-*α*, an inflammatory mediator, may be responsible for the reduced colonic uptake of butyrate [113]. The deprivation of butyrate or any other SCFAs, in conjunction with the toxic metabolites derived from proteolytic fermentation when saccharolytic fermentation is not possible, has long been proposed for the pathogenesis of gastrointestinal disorders (or even cancer) identified to originate in the distal colon [114]. Similar proposal concerning their therapeutic role in the regulation of inflammatory immune responses and the defense of mucosal immunity with respect to cellular functions in the colon is also made [26, 115–117]. The therapeutic effects of either butyrate alone or combination of SCFAs on patients with moderate-to-active colonic inflammation were confirmed. Many of the UC patients showed responsiveness toward rectal enema treatment of butyrate (amid methodological and procedural differences), whereby symptomatic improvement was reported afterward and coincided with a reduction in the inflammatory parameters [93, 118, 119]. Despite the fact that clinical data have not established an efficacious dietary quantity/frequency of butyrate [120–122], current in vitro and ex vivo studies do implicate a regulatory role in intestinal mucosal immunity.

## 5. Genes and IBD

Disease expression observed in individuals with IBD are a result of genetic predisposition to mounting an inappropriate inflammatory response toward commensal microflora (i.e., anergy is breached) [123, 124]. Some view immunodeficiency phenotype as the principle drive behind IBD pathogenesis [125, 126], but external variables including degree of bacterial load, malnutrition, surgery, and/or use of immunosuppressant therapy must be present in order to facilitate the disruption of the mucus layer and/or epithelial tight junctions. As a result, rendering the submucosal compartments to become increasingly susceptible to bacterial exposure, penetration, and adherence [36, 127–130]. Most importantly, these variables predispose to abnormal interactions with the microbiota. Whether observed dysbiosis, particularly in CD, is a result of the host reaction and/or therapy or a precursor to disease development is unclear yet.

Early progress was centered on characterizing genetic variations in association with IBD susceptibility as supported by familial aggregation studies and population-based cohort surveys. It was suggested that geographic location, ethnic background, socioeconomic class, and positive familial IBD history (e.g., first-degree relatives and monozygotic twin) are all variables dictating the risk in an individual for developing IBD [131, 132]. Out of the 71 CD candidate genes and 47 for UC that have been identified to date [133], only about 30 of them are clearly delineated [50]. Genetic studies are providing more concrete evidence for earlier epidemiological studies, but at the same time pose additional questions that further highlight the complex etiologies associated with IBD. Despite some similar phenotypic traits, IBD subtypes do not share all susceptibility loci. Another key finding reveals that allelic variants to date confer to only a small fraction of disease heritability in the IBD populations. This suggests that as yet unidentified genes or other environmental factors are attributable to IBD pathophysiological development. Indeed in CD genetic predisposition to bacterial infections is generally not enough to bring forth the clinical symptoms. A number of additional environmental factors have now been delineated, and these include smoking (promotes CD and protects against UC) [134], appendectomy (may promote CD and protects against UC) [135], nonsteroidal anti-inflammatory drugs (promote CD) [134], bacterial or viral infections (disrupt mucosal permeability of the intestine) [134], and early exposure to antibiotics (promote CD) [136].

### 5.1. Mechanistic Model of Genetic, Nutrient, Microbial Interaction: Function of NOD2

The first and most consistent mutations associated with increased susceptibility of CD (but not UC) are in the nucleotide-binding oligomerization domain containing 2 (NOD2) gene located on chromosome 16q12. Formerly it was known as caspase activated recruitment domain protein 15 (CARD15) gene [39]. Considerable research has revealed a complex of interactive components necessary for the normal function of disposing the host of bacterial invaders. This section reviews the components, genetic and dietary, needed for such function. It is used primarily here as an example of the mechanistic interactive effects outlined above and how dysfunctions in different components could lead to disease.

Nod2 protein (product of the NOD2 gene) belongs to the family of PRRs. Upon recognition of bacterial-associated PGN patterns, it mediates the activation of two pathways—NF-**κ**B and mitogen-activated protein (MAP) kinase. This intracellular receptor located predominantly in Paneth and other cells situated in the distal ileum plays a key part in the innate immune defense by eliminating intracellular bacteria or bacterial debris [137]. Its genetic mutations confer susceptibility in CD mice models [138]. Three major NOD2 mutations associated with the LRR domain have been confirmed: two missense SNPs (Arg702Trp and Gly908Arg) and one frameshift variant (Leu1007fsinsC), respectively [139–141]. All three mutations share similar restricted activation of the NF-*κ*B pathway in response to LPS and PGN treatments [140, 142, 143]. Despite the prevalence of NOD2 mutations present among the Caucasian populations (approximately 30% of patients of European ancestry have at least one of the three polymorphisms), the genetic penetrance corresponds to less than 10% of CD manifestation found in the carriers [144, 145].

### 5.2. Genetic Components for Normal NOD2 Function

A number of genes interact to promote normal NOD2 function. These include genes controlling Toll-like receptors, autophagy genes (ATG16L1 and IRGM), and most recently, products of Transducin-like enhancer of split 1 (TLE1) also demonstrate major effects. Even though loss of function/regulation in NOD2 may not compromise NF-*κ*B signalling completely, an imbalance of immune activity among mucosal cells is often the case due to oversecretion of proinflammatory cytokines as an attempt to dispose bacterial components [146]. A recent paper reported that TNF receptor 4 (TRAF4) is responsible for downregulating the activation of NF-*κ*B, hence limiting the innate response. This indicates that mutations in this downregulator may be key in correcting the acute innate response similar to how bacterial inoculation could do for NOD2 polymorphisms [147].

Autophagy is a highly conserved cellular process recognized for its role during starvation and in intracellular pathogen clearance. In the former, intracellular components are degraded indiscriminately to ensure cell viability. In the latter case, the process involves the precise rearrangement of intracellular constituents (e.g., bacteria, mitochondria, intracellular membranes, and proteins) to form a macroautophagy structure in order to isolate the foreign pathogen for digestion. It is then sequestered in a double-membrane cytosolic vacuole called an autophagosome which later fuses with lysosomes for further processing [148, 149]. Genome-wide association (GWA) studies have identified two sequence variants involved in the autophagy pathway, ATG16L1 and IRGM1, which confer to the genetic susceptibility of CD [150–153]. Although the functional consequences as to how ATG16L1 and IRGM1 mutations contribute to the pathogenesis of CD are not fully understood, accumulating human genetic data suggest that the location of ATG16L1 risk allele on chromosome 2q37 might be linked to autophagy mutations found in macrophage and Paneth cell.

Most recently a number of proteins have been identified in vitro to interact with NOD2 [154]. Using a yeast 2-hybrid screen some have been connected to a gene TLE1 which affects mucin biosynthesis and apoptosis. These epistatic interactions are putatively regulatory, and mutations in one of the alleles of TLE1 appear to be necessary for CD risk in the presence of classical NOD2 mutations. This allele may also increase the risk for UC which is independent of NOD2 mutations.

### 5.3. Nutrient Components for Normal NOD2 Function

In addition to the genetically mediated controls outlined for NOD2, two environmental variables have been shown as requirements for normal execution of intracellular bacterial elimination. One study by Wang et al. linked in vitro 1,25-dihydroxyvitamin D (or vitamin D) requirement for normal NOD2 function which was measured through the release of stimulated NF-*κ*B products and defensin *β*2 [155]. The presence of mutations of NOD2 could not be corrected by increasing media levels of vitamin D. The other paper by Leung et al. reported that, in response to the selective modulation of histone acetylation in the NOD2 promoter region by butyrate, an upregulation of Nod2 was observed. The result is a dramatic enhancement in the production of two chemokines, IL-8 and GRO-*α*, in the presence of PGN. However, in its absence, butyrate only had a slight effect on IL-8 concentration without altering the NF-*κ*B associated IL-8 promoter region concentration levels. Their results are in agreement with the observation made by Fusunyan and colleagues, such that NF-*κ*B suppression by butyrate is an indication that the upregulation of IL-8 must be independent of NF-*κ*B-mediated mechanism [78, 100]. Butyrate addition to the in vitro Caco-2 cell line enhanced PGN-mediated IL-8 and GRO-*α* production. These products also depended on the induction of NF-*κ*B as well as PGN [78]. Taken together these two reports outline a molecular model for the interactions between the NOD2 genetic consortium and 2 important environmental variables which impact on normal function. To date there is no information to our knowledge whether these 2 variables, vitamin D and butyrate, serve redundant or synergistic (additive) functions. Until that time the role of butyrate may be essential for appropriate clearance of intracellular bacterial products and innate immunity.

## 6. Dietary Carbohydrates, Symptoms, Pathogenesis

The impact of dietary interventions for the management of IBD has kept abreast of the scientific research outcome in the last two to three decades, albeit that results are less compelling than theory would suggest. Rationales for specific interventions in particular are more defined. For example, the use of anti-inflammatory omega-3 fatty acids seems rational, although outcomes are not satisfactory [172, 173]. In the case of carbohydrates, Gibson and Shepherd argue that distribution and subsequent rapid fermentation of FODMAP molecules predispose the distal small intestinal and colonic lumen to increased intestinal permeability, an underlying factor to the development of CD in genetically susceptible individuals [8]. They have advocated the pathophysiological involvement of FODMAPs in CD as a direct consequence of widespread consumption in Western societies. Excessive exposure of high fructose corn syrups and caloric sweeteners, commonly present in soft drinks and various manufactured food products [174], also appear to correlate with an increase in functional GI symptoms. As well, lactose sensitivity, independent of known genetic lactase status, has now been confirmed in patients with CD [160].

Consumption of FODMAPs exerts osmotic effects by increasing luminal fluid, inducing intestinal distension, altering intestinal contractile patterns, and accelerating transit time [175]. Development of these symptoms leads to the concept of global restriction of all poorly absorbed, rapidly fermentable short-chain carbohydrates as opposed to selectively limiting a few food items [176–178]. FODMAPs aggravate symptoms possibly further by inducing abnormal motility patterns as a consequence of colonic microfloral modification to accommodate the high volume of such consumption [163] or the incompletely evaluated role of intestinally released gut hormones as described with the prebiotic lactulose [179, 180].

### 6.1. Effects of Carbohydrate Withdrawal on Microbial Flora

Early etiological studies of IBD (especially CD) have consistently suggested that high consumption of refined sugar may be an independent risk factor [181–185]. More recent publications, however, have questioned this effect [186–188]. Nevertheless, a possible explanation for this observation has been provided by the proposed prebiotic concept [2]. There are, however, little data on microbial effects of complex carbohydrate withdrawal. Rats restrictive of food for 20 weeks resulted in nonsignificant changes in reduction of total anaerobic microbes and no significant shifts in population species [189]. When rats were fed sucrose or starch in equicaloric amounts for 9 months, no weight changes occurred, but the aerobic population increased and ratio of anaerobes to aerobes decreased [190]. Most importantly the total SCFAs production was significantly higher in starch than sucrose-fed rats, although the ratios remained the same.

### 6.2. Effects of Carbohydrate Feeding on Microbial Flora

On the contrary there are abundant data on the effects of poorly digested carbohydrates on microflora. Maldigested carbohydrates in general alter numbers [191] and type of intestinal cells [192], SCFAs production, colonic pH [191, 193], and microbial numbers as well as diversity [194]. As for prebiotics (those maldigested carbohydrates which fit more to the definitions as proposed by Gibson et al. [195]), short-chain (oligofructose) as well as long-chain fructose (inulin) polymers, all of which promoted the production of SCFAs [196], Bifidobacteria and Lactobacilli species in stool [197, 198], and other mucosal-associated microbial species [199].

In the case of IBD, the introduction of fructooligosaccharides or lactulose in healthy rats has demonstrated a combined effect of increased bacterial translocation, epithelial cell proliferation, colonic epithelial injury, and mucin production despite prebiotic consumption [200]. Among rats fed a FODMAP-like diet in conjunction with *Salmonella* species infection, severe colitis developed while only mild colonic inflammation was observed in controls [200].

Furthermore, a number of other published studies have demonstrated the protective role of both traditional prebiotics as well as other maldigested carbohydrates against experimentally-induced colitis (reviewed in [5]). The animal models employed in those experiments include the IL-10-deficient and trinitrobenzene sulfonic acid (TNBS) mouse model of CD and the DSS mouse model of UC. In these cases lactulose, fructo-oligosaccharide, and trans-galacto-oligosaccharide prebiotics as well as germinated barley foodstuffs (derived from beer production) alter colonic physiology via pH, SCFAs production, microbial species, and outcome of induced colitis.

Probiotics and prebiotics have generally been associated with improvement in clinical IBD [201]. It is postulated that pro- and prebiotics modulate the extent of inflammation during the progressive stage of the condition. In this context probiotics may have an advantage in UC [202], despite benefit of any specific probiotic in CD to date has not been substantiated [203]. Prebiotics in CD have generally shown some effect but again not substantiated (see the following).

### 6.3. Irritable Bowel Syndrome and Inflammatory Bowel Disease

An example where dietary intervention takes into consideration both outlined concepts of carbohydrate effects is IBS. Neurological disturbances [204–207], abnormalities in the brain-gut axis [208, 209], hyperreactivity to stress [210], and impaired gut motility or transit [211, 212] are etiological factors previously proposed to drive symptom profiles of IBS. However, until recently etiological explanations have begun to resemble those of IBD. Genetic factors [161], altered enteric microbiota [164], with a variation of additional bacterial overgrowth in the small intestine [33], and the role of host-microbial communications are gaining importance [6, 7, 162, 165, 213]. High production of acetate and propionate have been observed in correlation with more severe IBS symptoms in patients as reported by Tana et al. [214]. Response to selective probiotics in IBS has also been reported albeit with variable success [10]. While there may be some increased inflammatory cells found on histopathology [166, 215], in cases of postinfection, there is no tissue destruction as seen in IBD.  Table 3 outlines some of these similarities.

In general, symptoms in active IBD are attributed to inflammatory processes. However, a fraction of patients defined by clinical criteria to be in remission, nevertheless suffer symptoms which are reminiscent of IBS and satisfy Rome II or III criteria [216]. The use of classical anti-inflammatory medication (e.g., corticosteroids, immunomodulators, etc.) does not seem to alleviate these symptoms and may affect up to a third of patients [216]. Nonetheless, evaluation of fecal calprotectin (a protein marker of true inflammation) in such patients shows elevated levels supporting the notion that these IBS-like symptoms may also be mediated by inflammation. Generally, calprotectin levels are expected to be normal in classical IBS [217].

At present, therapeutic developments targeting those factors remain a complicated task due to the heterogeneity within and among individuals. Unlike IBD, which has a defined immunological pathology, IBS is a highly subjective disorder where hypersensitivity to foodstuffs is mistakenly perceived by patients as the primary symptomatic factor (the common ill-perceived food constituents are ones originating from dairy products, fructose and wheat products) [167, 168, 208]. Carbohydrate ingestion, in particular, are often avoided. There are thus two approaches to reduce the symptoms as a result of carbohydrate ingestion. Both will be discussed in the following.

## 7. Concepts of Carbohydrates and Therapy: FODMAP Withdrawal Approach

### 7.1. Irritable Bowel Syndrome

After having incorporated FODMAPs as part of their daily diet, subjects (those with preexisting IBS, quiescent IBD condition, or free of intestinal diseases) across several studies had all experienced an increase in effluent load, diarrhea secondary to altered bowel/motility movements, and an overall exacerbation of abdominal symptoms (i.e., flatulence, pain, and bloating) [169, 178]. Contrarily, results derived from other studies involving the restriction of one or more FODMAP food items all showed an improvement of abdominal symptoms in IBS patients [170, 218]. 

Twelve participants who had previously undergone ileostomy were subjected to either a high or low FODMAP diet for a 4-day period [178]. A 20% increase in the ileal effluent was observed after the participants consumed a high FODMAP diet compared to low, taken into account water content and dry weight also. The effluent consistency was reportedly thicker for the low FODMAP diet as opposed to the high FODMAP diet. Such changes to the nature of ileostomy output are likely influenced by the osmotically active FODMAP components. 

Isolated fructose restriction for IBS patients with fructose malabsorption also demonstrated a sustained improvement of functional gut symptoms [218]. In a randomized placebo-controlled crossover trial, fructose, fructans (these are linear or branched polymers of fructose) and a mixture of the two substrates were randomly reintroduced to the original low FODMAP test diet given to a group of fructose malabsorbers with some form of known IBS condition [169]. Despite responding well to the low FODMAP diet for the 10-day duration, 70% of these patients reported symptom recurrence (i.e., diarrhea, abdominal pain, wind, bloating, etc.) upon having their daily meal challenged with fructose and/or fructans in a dose-dependent manner compared to only 14% who received glucose (control). In addition, fructose and fructan combined promoted the greatest symptom severity than either substance alone. This study further supports the dietary principles of FODMAP withdrawal and demonstrates how eliminating the right dietary component is critical to correct IBS symptoms. 

It is postulated that the many symptoms (especially diarrhea) felt by IBS patients may be more related to abnormal colonic fermentation rather than osmotic effects, possibly a result of antibiotic- or gastroenteritis-induced dysbiosis [163]. One experiment assessed such correlation by measuring the total body excretion of hydrogen and methane gas in a 24-hour calorimetric test [219]. A comparison between healthy and symptomatic IBS subjects, each consuming two types of diet—a standard fiber-rich and fiber-free diet—found that a significant improvement in abdominal symptoms is in fact associated with the reduction of gaseous products from fiber-free consumption. 

Ong and colleagues conducted a randomized, single-blinded, crossover trial to evaluate the impact FODMAP consumption has on the extent and spectrum of intraluminal gas production in 15 healthy volunteers compared to 15 IBS patients by Rome III criteria [220]. Breath hydrogen excretion levels remained fairly high in both groups after a 2-day high FODMAP diet. They observed that those subjected to a high FODMAP diet have a significantly higher incidence of symptoms associated with luminal extension. Interestingly, those without IBS criteria also reported increase in gas production when subjected to a high FODMAP diet, but it did not translate to IBS-related symptoms [220]. Thus, these results indicate that FODMAPs do not cause IBS but that symptoms are triggered by the exaggerated bowel response to gaseous distension [169, 220]. Another study from the UK confirmed the benefit of a low FODMAP diet in IBS patients [221]. Staudacher et al. conducted a diet questionnaire in 82 patients with IBS where they were roughly divided into equal proportions to consume either a standard or a low FODMAP diet. Both groups showed significant improvements in the overall and specific symptoms (e.g., bloating).

### 7.2. Inflammatory Bowel Disease

In the case of IBD, little information is available concerning the specific trials involving carbohydrate restriction. The use of elemental/enteral diets particularly in children to induce CD remission has been explored, but it involves the restriction of most elements from reaching the lower intestine [222, 223]. A randomized controlled trial of carbohydrate restriction was reported by Lorenz-Meyer et al. after 15 years of study [224]. They found some benefit to prevention of relapse in patients with CD, but intention to treat analysis failed to reach significance. More recently, FODMAP withdrawal was reported in a pilot study of 72 patients (52 CD, 20 UC) over a 3-month period [225]. Out of about 70% diet-adherent patients, 50% responded favourably with reductions in abdominal symptoms.

## 8. Concepts of Carbohydrates and Therapy: Emphasis on Prebiotics

### 8.1. Irritable Bowel Syndrome

In contradistinction to FODMAP withdrawal diet, regular consumption of single or mixtures of prebiotics has also been explored for IBS in a few studies. The concept that symptoms of carbohydrate intolerance in healthy persons can be overcome by regular short-term ingestion was observed in populations with lactose intolerance [246]. A formal randomized crossover study of lactose feeding in lactose maldigesters demonstrated both symptomatic and fecal microfloral adaptation [247]. Although symptomatic improvement of lactose intolerance may be due to a placebo effect [248], changes in hydrogen and fecal bacteria are physiological [249–251].

While it is well recognized that prebiotics induce symptoms in patients, there are now two controlled trials in patients with IBS which demonstrated symptomatic “adaptation” to prolonged feeding. Paineau et al. published a double-blind randomized controlled trial using short-chain fructo-oligosaccharides in 105 patients and reported a global, yet highly specific, symptomatic improvement by the end of the 6-week trial [252]. Similarly, trans-galacto-oligosaccharides employed by Silk et al. in a crossover trial of 44 patients over 12 weeks also reported global and specific improvements [253]. These two studies demonstrate that it is possible to improve symptoms in IBS simply by providing prebiotics on a continual basis. It is not, however, clear whether such improvements were due to “psychological adaption” or bacterial adaptation to carbohydrates.

### 8.2. Inflammatory Bowel Disease

Several studies examining the possible benefits of classical prebiotics (fructose or galactosyl polymers) and poorly digested fibers (e.g., Ispaghula husk, germinated barley foodstuffs) to IBD have been published. The rationale as outlined rests on their ability to modulate the intestinal microflora and their beneficial consequences associated with SCFAs production [91, 206]. These studies comprised of 744 patients with UC, CD, or P (postoperative ileoanal anastomotic pouch inflammation). The variety of indications is described in Tables 4(a) and 4(b), and includes maintenance of remission [226, 227, 231, 234], mild to moderately active disease [91, 228–230, 232, 236–240, 244], prevention of postsurgical CD recurrence [226, 242], and physiological assessment of adaptation capability [233]. The studies include 8 randomized controlled trials of which 3 were double blinded [237, 238, 244] and two were crossover design [226, 238]. The studies extended from 2 weeks to 24 months (mean 4.8 ± 6.1 months, with a median of 1.6 months). A total of 510 patients were treated with active agent and 234 were controls. Of the controls 31 patients received probiotics without prebiotics [241]. Forty-nine treated patients were crossed over to placebo [226, 238]. While endpoints varied, only two studies failed to show benefit. Six of the randomized studies (4 for UC in remission [226, 227, 241], one active CD [244], and one active P [238]) showed better or nonsuperior remission rates for UC, also improvement in clinical score for CD or P. A small study showed reduction of proinflammatory cytokines in UC [230]. However, the studies failing to show benefit included the largest and most carefully conducted DBRCT (double-blind randomized controlled trial) of patients with active CD [237]. Importantly, it also included the only study albeit observational, evaluating the role of synbiotics in CD postsurgery recurrence [242]. Additional well-conducted trials are needed to lend clinical credence to effective use of prebiotics in IBD. 

## 9. Summary and Conclusions

The basic premise of this paper is a conceptual contrast of the rationale of either using a select group of prebiotic molecules to alter microflora and microbial metabolism or to withhold a wide array of carbohydrates which includes those prebiotics. The emphasis of these interventions is on use in IBD, but IBS is used as a clinical model to outline available but to date limited number of trials to show symptomatic efficacy. The two principles pose a scientific conundrum particularly in IBD, while there is evidence that bacterial immune interactions play a significant role in IBS abnormal immune response in IBD lead to tissue destruction. 

There is limited evidence that both approaches (withhold FODMAP entirely or use selective parts of FODMAP) in IBS result in symptomatic improvement in a significant percentage of patients within a certain time frame. The use of prebiotics in IBD is not settled in either active or remitting disease. Information on the use of FODMAP or general carbohydrate withdrawal, to our knowledge, has been limited with IBD. The IBS-like symptoms in IBD may be related to intestinal inflammation making its pathogenesis similar but different from that in true IBS. As such the role of beneficial bacteria and SCFAs may be more important in the former.

The real “conundrum,” then, is whether the additive or withdrawal approach can induce microbial changes which subsequently lead to amelioration of symptoms (as in IBS or IBS-like symptoms in IBD), but also modulation of the immune response especially inflammation. If both approaches affect the microflora, what organisms are (equally?) modulated by a reduction in specific nutrition as well as kept in check by other organisms like lactic acid-producing bacteria? There is limited research on effects of withdrawal (whether total nutrient or specific nutrients like carbohydrates). There are many publications on effects of addition of prebiotics or complex fibers. The example of NOD2 suggests that certain dietary components may be necessary for normal function, but redundant functions are likely. Nevertheless, until more information is available, a judicious use of the discussed approaches and time of use should be considered for symptom control, with withdrawal (the less tried approach) for IBD.

## Figures and Tables

**Box 1:**
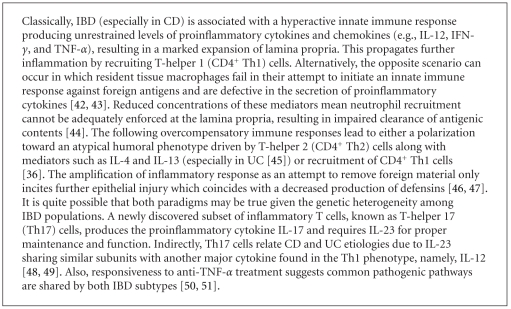
IBD pathogenesis.

**Table 1:** List of poorly digested carbohydrates comprised of FODMAP and select prebiotics (∗), as well as their respective sources. This is not a complete list, and other complex carbohydrates which have effects on bacteria are also included in FODMAP.

Molecular form	Common sources
Inulin (∗)	Onions, leeks, chicory, artichoke, wheat, banana
Oligofructose (∗)	Hydrolysis product of inulin
Short-chain fructo-oligosaccharide (∗)	Hydrolysis product of inulin
Trans galacto-oligosaccharides (∗)	Manufactured from lactose
Lactulose (∗)	Manufactured from lactose
Fructo-oligosaccharides (∗)	May be present in breast milk, formed from lactose
Isomalto-oligosaccharides (∗)	Present in foods, potential prebiotic
Lactose (∗)	Present in dairy products made from animal sources, prebiotic mostly in lactose maldigesters
Polyols	Sugar alcohols (sorbitol, mannitol, xylitol, maltitol, and isomalt), cauliflower, avocado, mushrooms

**Table 2:** Some immunoregulatory functions of butyrate.

(i) Increases choline acetyltransferase immunoreactive (ChAT-IR) enteric neurons in vivo and in vitro (ii) Increases cholinergic-mediated colonic motility and contractile response ex vivo	[72]

(i) Modulates oxidative stress in healthy colonic mucosa (ii) Promotes glutathione (GSH) and lower uric acid concentrations compared	[73]

(i) Promotes the differential expression of 500 genes in human colonic mucosa (ii) Increases gene expression of transcriptional regulation pathways: fatty acid oxidation, electron transport chain, and oxidative stress (iii) Increases gene expression related to epithelial integrity and apoptosis	[74]

(i) Influences colonic function, mainly by histone deacetylase inhibition	[75, 76]

(i) Reduces inflammatory responses in vitro, mainly by inhibition of NF-*κ*B activation	[77]

(i) Mediates NOD2-dependent mucosal immune responses against PGN	[78]

(i) Modulates an intracellular JAK/STAT1 signaling cascade which inhibits NO production	[79]

(i) Enhances upregulation/detection of PRRs on intestinal epithelial cells	[80–83]

(i) Anticarcinogenic/angiogenic by modulating the activity of several key regulators involved in apoptosis and cell differentiation	[84–86]

(i) Enhances colonic defense barrier	[87–89]

**Table 3:** Comparison of putative pathogenic mechanisms in inflammatory bowel disease (IBD) and irritable bowel syndrome (IBS).

IBD	(i) Genetic predisposition (extensive)	[39, 131, 140, 150, 152, 156, 157]
	(ii) Intestinal microflora alterations	[55–57, 59–61, 65, 124, 158, 159]
	(iii) Altered immunity (extensive)	[42–44, 123, 125, 126]
	(iv) Altered carbohydrate sensitivity	[160]
	(v) Tissue destruction and complications	[50, 124]

IBS	(i) Genetic predisposition (exists but not yet worked out)	[161, 162]
	(ii) Microflora alterations especially after gastroenteritis	[6, 33, 163–165]
	(iii) Altered immune response (variable and mild)	[166]
	(iv) Altered carbohydrate sensitivity	[167–170]
	(v) No evidence for tissue destruction	[171]

**Table:** (a)

Disorder	*N* = patients	Study type	Active agent	Outcome	Reference
UC^1^	29	RCT	Ispaghula husk	Improved	[226]*
UC^1^	102	RCT, OL	Plantago Ovata	Nonsuperior	[227]
UC^2^	10	OL	GBF	Improved	[228]
UC^2^	18	OL	GBF	Improved	[91]
UC^2^	21	OL	GBF	Improved	[229]
UC^2^	40	RCT	GBF	Cytokine decreased	[230]
UC^1^	59	RCT, OL	GBF	Lower recurrence	[231]
UC^2^	19	OL	OFS + IN + Bif	Improved clinical endoscopy	[232]
UC and CD^1^	20 (10 controls)	OL	Lactulose	Adaptation in UC, but not in CD	[233]
UC and CD^1^	31	OL	Lactulose	No effect, but improved quality of life in UC	[234]
CD^2^	10	OL	FOS, IN	Improved score	[235]
CD^2^	10	OL	FOS, IN	Improved	[236]
CD^2^	103	DBRCT	FOS	No clinical benefit, despite impacting on DC function	[237]
P^2^	20	DBRCT	IN	Improved inflammation	[238]*
P^2^	21	OL	Lactose	Decreased bacterial sulfomucins	[239]

**Table:** (b)

Disorder	*N* = patients	Study type	Active agent	Outcome	Reference
UC^2^	16	OL	OFS + IN + Bif	Improved clinical endoscopy	[240]
UC^1^	120	RCT	Bif/Psy/Bif + Psy	Improved quality of life with Bif + Psy	[241]
CD^3^	30	OL	Mixed fiber + IN + 4 Lacto	Failed to prevent relapse	[242]
CD^2^	10	OL	Psy + Bif + Lacto	Clinical improvement	[243]
CD^2^	35	DBRCT	OFS + IN + Bif	Clinical improvement	[244]
P^2^	10	OL	OFS, Lacto	Improved and remit	[245]

RCT: randomized controlled trial; DBRCT: double-blind randomized controlled trial; OL: open labeled; GBF: germinated barley foodstuffs; FOS: fructo-oligosaccharides (<5 degrees of polymerization); OFS: oligofructose (5–10 degrees of polymerization); IN: inulin (<200 degrees of polymerization); Psy: psyllium; Bif: Bifidobacteria species; Lacto: Lactobacillus species.

*Crossover design,

^1^Disease in remission,

^2^Active disease,

^3^Maintenance after surgery.
